# Recurrent Post-viral Rhabdomyolysis: A Case Report

**DOI:** 10.7759/cureus.70901

**Published:** 2024-10-05

**Authors:** Alyssa Breedlove, Ashton Rohrschneider, Richard Virgilio, John R Fleming

**Affiliations:** 1 Clinical, Biomedical, and Educational Research, Edward Via College of Osteopathic Medicine, Spartanburg, USA; 2 Clinical, Biomedical, and Educational Research, Edward Via College of Osteopathic Medicine, Auburn, USA; 3 Clinical Affairs, Edward Via College of Osteopathic Medicine, Auburn, USA; 4 Family Medicine, Edward Via College of Osteopathic Medicine, Spartanburg, USA

**Keywords:** covid-19, emery-dreifuss muscular dystrophy, idiopathic rhabdomyolysis, non-traumatic rhabdomyolysis, post-influenza, viral-induced rhabdomyolysis

## Abstract

Rhabdomyolysis is a relatively rare condition caused by the damage and release of myocyte contents. It occurs most commonly secondary to strenuous exercise. Rhabdomyolysis carries the risk of life-threatening negative sequelae such as acute kidney injury or death. This case report describes a middle-aged male patient who has presented with rhabdomyolysis five times over the past nine years, each following a viral illness. No inborn errors of metabolism, neuromuscular junction conditions, or myopathies were found to explain the patient's recurrent rhabdomyolysis except for two variants of unknown significance in the SYNE2 gene that has been linked with Emery-Dreifuss muscular dystrophy.

## Introduction

Rhabdomyolysis occurs when the intracellular contents of muscle cells are released into the bloodstream following the necrosis and breakdown of the sarcolemmal membrane of the skeletal muscle tissue [1,2]. The pathophysiology of this disease process appears to occur due to an increase in intracellular calcium, which degrades the myocyte, resulting in the release of the intracellular contents into the extracellular space [3]. Rhabdomyolysis can present in any type of patient, although some studies suggest that it may be more common in males, African Americans, people with a BMI >40, and children under 10 or adults over 60 years of age [1]. It most commonly occurs after strenuous exercise, but can also present after status epilepticus, neuroleptic malignant syndrome, ischemia, drug reactions, inherited myopathies and enzyme deficiencies, and traumas like electrical shocks, crush injuries, and burns [3]. The medication most commonly associated with rhabdomyolysis are statin drugs with several proposed mechanisms related to the skeletal muscle cell membrane integrity, coenzyme Q10 deficiency, and abnormal signal transduction [3]. 

Patients with rhabdomyolysis can present with a variety of symptoms, ranging from completely asymptomatic to symptoms as severe as acute renal failure and hypovolemic shock. However, the most common presenting symptoms include muscle pain, weakness, and a red-brown discoloration of their urine [2]. Diagnostic criteria include serum creatine kinase (CK) >1000 or at least five times the normal value [1]. Patients may also present with other lab abnormalities like elevated serum myoglobin, myoglobinuria, electrolyte abnormalities, and elevated serum creatinine [1]. 

Once diagnosed, rhabdomyolysis should be treated with aggressive fluid resuscitation and removal of the offending cause of the muscle injury [1]. Studies have shown that a breakdown of 100 g of muscle tissue or greater releases enough myoglobin to cause renal injury [3]. Myoglobin is nephrotoxic and leads to tubular epithelial cell damage which vasoconstricts the afferent arterioles of the kidney, resulting in a decrease in glomerular filtration, an increase in serum creatinine, and a decreased ability to filter myoglobin [4]. Therefore, aggressive intravenous (IV) fluid resuscitation up to 1.5 L/hr is recommended to increase diuresis and dilute the levels of toxins in the blood [4]. 

Patients who are young at presentation or who have recurrences may undergo genetic testing, muscle biopsy, and the forearm ischemic exercise test to evaluate for potential myopathies, metabolic disorders, and muscle dystrophies [4]. Symptoms that could indicate a potential metabolic disorder include the involvement of all muscles, including the neck, jaw, and paraspinal muscles, and can be exacerbated by fasting and stress [5]. Carnitine palmitoyltransferase 2 (CPT2) is the most common disorder of fatty acid oxidation and has documented association with recurrent rhabdomyolysis, especially with certain triggers like fever, infection, statins, and exposure to temperature extremes [5]. 

A review of the existing literature reveals other cases of post-viral rhabdomyolysis following COVID-19 infection, influenza A infection, coxsackie B virus, echovirus 9, and herpes simplex virus [6-10]. Other studies reveal a potential correlation between muscular dystrophies and recurrent rhabdomyolysis [11]. This case report explores a variety of potential causes of rhabdomyolysis in a patient having five separate incidents following viral illnesses. 

## Case presentation

A 45-year-old male with a past medical history of hypertension, obstructive sleep apnea, hyperlipidemia, and obesity presented to his primary care physician's (PCP) office in 2015 with one day of fevers, chills, and myalgias greatest in his upper legs. He tested positive for influenza and was treated with oseltamivir. Several days later, his myalgias had worsened and he had dark-colored urine and difficulty urinating, so he returned to his PCP who checked a CK level which returned >100,000 (normal range: 40-170). His PCP then directly admitted the patient to the hospital where he was treated with IV fluids and pain management inpatient for seven days. His CK levels were trended and returned to normal about 4-6 weeks after hospital discharge. 

In 2019, the patient presented similarly to his PCP's office with fever and myalgias in his legs. His PCP checked his CK level which peaked at 216,191; and again, the patient was admitted for inpatient IV fluids and pain management. He did not receive oseltamivir for this influenza infection as he presented more than 72 hours after the onset of symptoms, but he was admitted to the hospital for more IV fluid resuscitation and pain management. During this hospitalization, the patient's atorvastatin was discontinued at this time as this was believed to be the cause of his rhabdomyolysis. His CK returned to baseline about four weeks following hospital discharge. 

In 2022, the patient developed body aches, fever, myalgias, fatigue, and chills and presented to the emergency room where he was diagnosed again with influenza. His CK level was checked again and peaked at >100,000, along with elevated liver function tests (LFTs) and myoglobinuria. A thorough history from the neurologist consulted at that time revealed tingling, fasciculations, and muscle cramps in his legs about an hour after physical activity which did not present until after his first episode of rhabdomyolysis in 2015. Neurology ordered a deltoid muscle biopsy at that time due to suspicion for CPT1 or CPT2 deficiency or another inherited enzyme deficiency. The muscle biopsy performed following this hospitalization revealed no inflammatory myopathy, no vasculitis, no chronic dysmorphic changes, and no morphologic or histochemical changes. The only significant finding on muscle biopsy was a mild variation in muscle fiber diameter. Genetic testing revealed two variants of unknown significance (VUS) on the SYNE2 gene which is a gene that has previously been connected to Emery-Dreifuss muscular dystrophy type 5 (EMD5). The two variants in the SYNE2 gene were c.13883T>C (Leu4628Pro) and c.16234G>A (Ala5412Thr). 

In early 2024, the patient presented to the emergency room complaining of leg pain and dark-colored urine. In the emergency department, the patient's CK was low at 7 and the patient was discharged. The patient presented to his PCP the following day where his CK peaked at >22,000. This fifth episode of rhabdomyolysis occurred about three days following the resolution of symptoms from a COVID-19 infection. Following this episode of rhabdomyolysis, the patient was referred to a neuromuscular disorder specialty clinic. At this specialty clinic, the patient underwent electromyography testing which revealed normal results and further lab analysis which revealed a mildly elevated erythrocyte sedimentation rate of 27 (normal range: 0-22) and a mildly elevated gamma globulin at 1.7 (normal range: 0.6-1.6) with small monoclonal IgA kappa heavy chain without corresponding light chain. This result is consistent with biclonal gammopathy of undetermined significance, early myeloma, and amyloidosis and requires further evaluation with 24-hour urine monoclonal protein studies which are pending at the time of this report. 

Finally, in mid-June 2024, the patient presented again to the emergency room at the instruction of his family physician because of symptoms of dark urine and thigh and buttock pain about a week following a viral-type illness of fever, chills, and cough. The patient was admitted to the hospital following an elevated CK of 54,000 in the emergency room and treated with aggressive IV fluids for two days. On the day of discharge, the CK was elevated even further to 85,000, but the patient reported a resolution of symptoms and requested to go home. During this hospitalization, the patient did not undergo any further imaging or diagnostic tests to determine the underlying cause of his recurrent rhabdomyolysis. 

## Discussion

Rhabdomyolysis causes approximately 26,000 cases reported in the United States annually that is caused by the breakdown of muscle cells and release of toxic cellular material into systemic circulation [1,12]. While there are several known causes of rhabdomyolysis, like exercise, medication side effects, infection, and inherited conditions, there are still many unknown pathophysiological mechanisms underlying rhabdomyolysis. 

In this case report, we described a case of a patient who has had five episodes of rhabdomyolysis, each occurrence following a viral illness. The first instance of rhabdomyolysis in 2015 had no apparent cause and was thus considered to be idiopathic. Following the second episode of rhabdomyolysis in 2019, the patient's dose of atorvastatin was discontinued as statin drugs are a known cause of rhabdomyolysis [3]. The atorvastatin was not discontinued after the first episode of rhabdomyolysis as the patient had been on a stable dose for several years prior to that instance. 

The next instance of rhabdomyolysis in 2022 precipitated a more in-depth workup of potential causes. The patient was referred to neurology and to a genetic counselor to evaluate for muscular dystrophies, metabolic disorders, and other inherited conditions. Recurrence of rhabdomyolysis is most often associated with inherited metabolic muscle conditions [11]. Recurrent episodes following a viral illness are most commonly associated with CPT deficiency and other disorders of lipid metabolism [13]. The patient underwent a muscle biopsy, which revealed no disorders of the neuromuscular junction. The genetic panel revealed two VUS at c.13883T>C (Leu4628Pro) and c.16234G>A (Ala5412Thr) in the SYNE2 gene, which is associated with EMD5. Emery-Dreifuss muscular dystrophy (EDMD) presents as a triad of contractures, progressive muscle weakness and atrophy, and cardiac abnormalities like atrial tachyarrhythmias and cardiomyopathy [14]. The SYNE2 gene associated with EMD5 is responsible for the Nesprin-2 protein which is involved in nuclear envelope localization and structural integrity of myocytes [13]. Interestingly, EDMD presents with baseline mildly elevated CK levels which is believed to be due to chronic rhabdomyolysis [15]. We propose that the chronic rhabdomyolysis could potentially be associated with EDMD and was exacerbated by the viral illnesses that the patient contracted. In a meta-analysis, the pooled prevalence of EDMD in all age groups was 0.39 per 100,000 [16]. While EDMD has a variety of forms depending on the inheritance pattern of the genes, they all typically have similar symptoms, which usually begin in the first or second decade of life. However, the disease has shown significant variability with regard to age at onset, severity, and progression as fatty acid oxidation disorders and muscular dystrophies are considered to be a spectrum of disorders [17]. There have been cases observed of adult onset with slow progression, which is potentially what is seen in this patient. 

This case is puzzling as each occurrence of rhabdomyolysis in this patient followed a viral illness. Unlike inborn errors of metabolism or other genetic conditions, the patient did not present with recurrent rhabdomyolysis as a child or young adult. Moving forward with the recommendations of genetic counseling and neurology, the patient has been instructed to be proactive with all annual vaccinations, including influenza, COVID-19, and respiratory syncytial virus (RSV). The patient should also be asked about episodes of prolonged fasting prior to the development of his symptoms as this could point towards a fatty acid oxidation cause. With only the small genetic link to EDMD found to be a potential cause of this patient's recurrent episodes, there is no disease-modifying therapy available for the treatment of EDMD, only supportive measures. The principal concern in EDMD patients is death resulting from cardiac involvement; therefore, the patient should receive a cardiac workup of an echocardiogram and annual electrocardiogram (EKG) even if asymptomatic [18]. There is also a recommendation to avoid potential triggering agents of malignant hyperthermia in these patients, such as depolarizing muscle relaxants (e.g., succinylcholine) and volatile anesthetic drugs (e.g., halothane, isoflurane, desflurane, and sevoflurane) [19]. If the patient has children, with the two VUS that have potentially been passed down, his children should also stay proactive with their vaccinations and could also consider genetic testing. Further research into this variant of the SYNE2 gene could potentially reveal a definitive cause of this patient's recurrent post-viral rhabdomyolysis. 

Of note, the patient primarily reports only leg myalgias with no reported arm or back pain, which is an atypical presentation of rhabdomyolysis. 

## Conclusions

While this patient is still awaiting evaluation at a muscular dystrophy clinic at the time of this report, this case is unique in the fact of five recurrent rhabdomyolysis episodes following a viral illness with only a small genetic component linked to EDMD and a mildly elevated gamma globulin level as potential explanations. Further research into the two VUS identified in this patient could reveal a potential cause of the patient's recurrent rhabdomyolysis.
